# Late-onset unexplained epilepsy with dual amyloid and tau negativity: are alpha-synuclein seed amplification assays the next diagnostic step?

**DOI:** 10.1016/j.ebr.2025.100783

**Published:** 2025-06-13

**Authors:** Augustin Moreau, Elisabeth Ruppert, Frédéric Blanc, Olivier BOUSIGES, Benjamin Cretin

**Affiliations:** a Centre Mémoire, de Ressources et de Recherche d’Alsace (Strasbourg-Colmar), France; b Unité de Neuropsychologie, Service de Neurologie et hôpital de jour de Gériatrie, pôle de Gériatrie, Hôpitaux Universitaires de Strasbourg, Strasbourg, France; c International Research Center for ChronoSomnology (CIRCSom), Department of Neurology, Strasbourg University Hospitals, Strasbourg, France; d University of Strasbourg and CNRS, ICube laboratory, UMR 7357 and FMTS (Fédération de Médecine Translationnelle de Strasbourg), team IMIS/Neurocrypto Strasbourg, France; e Centre de Compétences des démences rares des Hôpitaux Universitaires de Strasbourg, France; f University Hospital of Strasbourg, Laboratory of Biochemistry and Molecular Biology, CNRS, Laboratoire de Neurosciences Cognitives et Adaptatives (LNCA), UMR7364, Strasbourg, France

**Keywords:** Late onset epilepsy, Cognitive decline, Dementia with lewy bodies, Cerebro-spinal fluid, Alphasynuclein, Seed amplification

## Abstract

Late-onset epilepsy of unknown etiology (LOEU) is associated with an increased risk of dementia. Current biomarkers, including cerebrospinal fluid (CSF) and positron emission tomography (PET) assessments for amyloid and tau, often fail to predict cognitive decline in a substantial proportion of LOEU patients. This case report presents a 67-year-old man with LOEU who later developed dementia with Lewy bodies (DLB). As cognitive decline progressed, emerging mild clinical features raised suspicion for DLB. Notably, cerebrospinal fluid analysis at this stage revealed negative amyloid and tau biomarkers but was positive for pathological alpha-synuclein using alpha-synuclein seed amplification assay (CSF ASyn-SAA). This finding highlights the potential clinical utility of CSF ASyn-SAA in achieving both earlier and more accurate DLB diagnosis. For LOEU patients exhibiting early signs of synucleinopathy, incorporating CSF ASyn-SAA into diagnostic panels could significantly improve diagnostic certainty, prognostic stratification, and opportunities for targeted therapeutic interventions. Further research is needed to investigate the yield of adding ASyn-SAA to CSF dementia panels in people with LOEU and progressive cognitive symptoms.

## Highlights

• LOEU increases the risk of dementia, which should be stratified as far as possible.
• Amyloid/tau biomarkers often miss incipient dementia with Lewy bodies (DLB).
• CSF α-synuclein assays may detect early DLB associated with LOEU.
• Early DLB detection enables targeted care alongside antiseizure drugs.

## 1 Introduction

Late-onset epilepsy of unknown etiology (LOEU) is increasingly attracting the attention from epilepsy specialists due to its significant risk of progression to dementia [1]. Current evidence suggests that Alzheimer’s disease (AD) is the most frequently implicated etiology, with LOEU patients often exhibiting neurodegenerative changes consistent with AD pathology. These changes include reduced CSF-Aβ42 and elevated CSF-tau levels, alongside cognitive impairments—particularly in memory, visuospatial abilities, and verbal fluency—and hypometabolism in the right posterior cingulate cortex and left precuneus [2]. Notably, LOEU patients with CSF-Aβ42 levels below the diagnostic cutoff face a substantially higher risk of progressing to AD (hazard ratio = 3.4 at 3-year follow-up) [3]. However, not all LOEU cases demonstrate AD-related pathological changes [4]. Furthermore, emerging data suggest a potential link between late-onset epilepsy, antiseizure medications, and Parkinson's disease or dementia with Lewy bodies (DLB) [[5], [6], [7], [8]]. These findings raise the possibility of an increased risk of subsequent synucleinopathy in LOEU [6,7]. Consistently, DLB has been reported in non-lesional late-onset epilepsy, though its exact prevalence is unknown [8]. In this context, a stratified approach to predicting the risk of cognitive decline seems necessary. This has become partially possible with the use of CSF or PET amyloid and tau biomarkers [1]. However, a significant proportion of LOEU patients remain unexplained, necessitating additional diagnostic tools [1]. Alpha-synuclein seed amplification assays (ASyn-SAA) were developed in 2016 and became commercially available in Europe in 2022 [9]. These assays accurately depict the cortical deposition of abnormal ASyn [[9], [10], [11]] and may therefore be particularly relevant for LOEU patients with dual amyloid and tau negativity, given the links between pathological ASyn and network hyperexcitability [12]. We present a case report demonstrating the potential of CSF-ASyn-SAA to broaden the detection of neurodegenerative disease to synucleinopathies in LOEU cases, potentially leading to earlier and more accurate diagnoses in the near future.

## 2 Case presentation

**Fig. 1:**
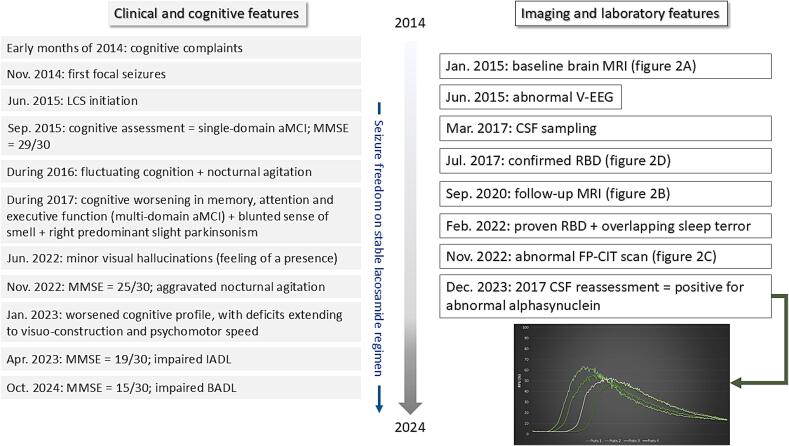
**clinical timeline of symptoms, signs and investigations.***Intended for color reproduction on the Web.* Legend: in green = amplification curves of abnormal alpha-synuclein in the patient’s baseline CSF (normal = no amplification or flat curves); BADL = basic activities of daily living; CSF = cerebrospinal fluid; IADL = instrumental activities of daily living; LCS = lacosamide, aMCI = amnestic mild cognitive impairment, MMSE = minimental state examination; MRI = magnetic resonance imaging; RBD = REM-sleep behavior disorder; V-EEG = video-EEG. (For interpretation of the references to color in this figure legend, the reader is referred to the web version of this article.)

**Fig. 2:**
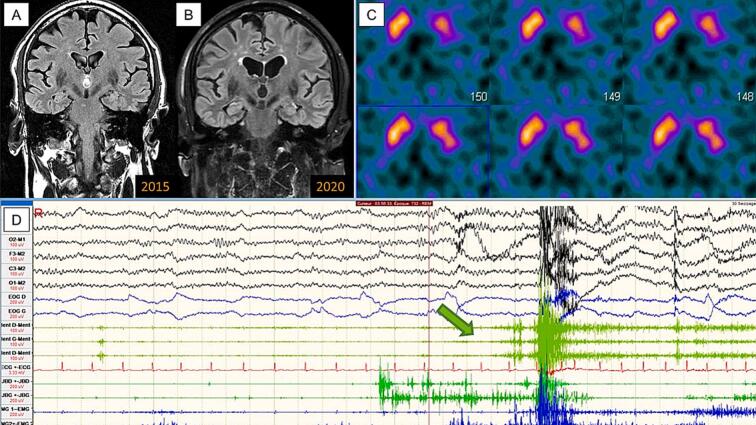
**selected paraclinical features.***Intended for color reproduction on the Web.* Legend: A-B = brain MRI, baseline and 5-years follow-up matched T2-FLAIR coronal slices showing progressive cortical and subcortical atrophy; C = predominant left-sided dopaminergic denervation on FP-CIT acquisitions (DaT-Scan); D = polysomnography, loss of muscular tone in REM-sleep (green arrow). (For interpretation of the references to color in this figure legend, the reader is referred to the web version of this article.)

The patient was a former nightclub owner, a right-handed 67-year-old French man, with a medical history of arterial hypertension, hyperlipidemia, coronary heart disease, and obstructive sleep apnea syndrome. He had no family history of neurodegenerative diseases. He attended our outpatient Neurology clinic for recurrent and stereotyped episodes, occurring up to 10 times per month (see timeline in Fig. 1). These episodes were non-motor onset focal seizures with impaired awareness, characterized by an aura of déjà-vu and/or nausea, dysgeusia, and epigastric rising. The ictal signs, reported by the patient’s spouse, included temporospatial disorientation, anterograde memory impairment, pallor, verbal automatisms, and occasional unresponsiveness. Each episode lasted 3–5 min on average (up to 15 min). Prior to the onset of seizures, he had noticed a subjective cognitive decline over several months, including forgetfulness, impaired attention, and word-finding difficulties. Video-EEG recordings revealed clear interictal epileptiform activity in the left temporal lobe without background slowing in the temporal, frontal or posterior regions. Notably, the overnight EEG was also normal, with no changes in sleep macro- or microarchitecture. Brain MRI was unremarkable (Fig. 2). Baseline neuropsychological examination revealed a preserved global level of cognition but mild cognitive impairment (MCI) with an isolated amnestic profile. The patient was diagnosed with LOEU and commenced treatment with oral lacosamide 100 mg twice daily.

Over the subsequent 10 years of follow-up, the patient remained seizure-free, but the gradually worsening clinical picture suggested a neurodegenerative condition, with progressive cognitive decline leading to dementia (Fig. 1). In March 2017, CSF analyses returned normal levels of amyloid and tau species, as well as other laboratory parameters. CSF samples were then frozen at −80 °C and stored for further analysis. From July 2017 onwards, the clinical and paraclinical data met the criteria for prodromal DLB [18], which was confirmed by the clinical outcome at the 10-year follow-up. When ASyn-SAA became available in our laboratory in 2023, the 2017 CSF samples were tested for pathological ASyn and the results were positive, consistent with a diagnosis of incipient synucleinopathy (Fig. 1).

## 3 Discussion

In this case report, the patient progressed not to AD but to isolated DLB, as demonstrated by accumulating clinical and paraclinical features (Fig. 1, Fig. 2). Neither the epilepsy syndrome nor the paraclinical workup – which included normal CSF amyloid and tau levels – could have predicted this outcome at baseline. Additionally, the patient’s initial cognitive phenotype was consistent with amnestic MCI, a presentation more commonly associated with temporal lobe epilepsy or early-stage AD [13] than with MCI with Lewy bodies (MCI-LB) [14]. If CSF ASyn-SAA had been available at baseline, its diagnostic utility would remain uncertain based on the current state of evidence. Two hypotheses can actually explain the relationship between the patient’s epilepsy and subsequent DLB. First, the seizures could have been an early manifestation of an incipient, isolated synucleinopathy that was initially below the ASyn-SAA detection threshold, resulting in a false-negative test. ASyn-SAA sensitivity indeed depends on cerebral synuclein load: it is high in case of neocortical DLB (whether limbic or diffuse), but is lower than 50 % and even 20 % in amygdala or brainstem predominant DLB, respectively [10,11]. Alternatively, epileptic activity and synucleinopathy could initially be independent processes, with DLB emerging later due to a proinflammatory state triggered by recurrent seizures. In this scenario, baseline ASyn-SAA would have been irrelevant and could have produced a false-positive result (specificity 77 %–100 %) [[9], [10], [11]]. In both cases, performing ASyn-SAA at epilepsy onset without clinical indicators of synucleinopathy would have been premature. Therefore, the assay was conducted more appropriately in 2017, when the patient’s MCI worsened and supportive features of dementia with Lewy bodies (DLB) emerged, including RBD, cognitive fluctuations, and visuospatial/executive deficits. If synucleinopathy had been confirmed at that time, the progression could have been anticipated more accurately. An earlier diagnosis would have allowed for a referral to a memory clinic, where personalized interventions could have improved the patient's quality of life. These interventions could have included diet and exercise recommendations, cholinesterase inhibitors, and cognitive and emotional support.

This case demonstrates that patients with LOEU can develop both AD and DLB. Whether focal seizures represent an early manifestation of neurodegenerative conditions, such as AD or DLB, warrants careful consideration. In AD, evidence suggests that cerebral amyloidosis may create a pro-excitatory state, which could explain seizures as a prodromal feature [15]. For DLB, although α-synuclein deposition correlates with network dysfunction, neuronal hyperexcitability, and seizures, a direct causal relationship remains unproven [12,16]. The frequent co-occurrence of LOEU and dementia may reflect a shared risk factor, which is advanced age, rather than a pathophysiological link [1]. Alternatively, it is conceivable that recurrent seizures might foster independent neurodegenerative processes (e.g., amyloid, tau, or synuclein pathology) through inflammatory mechanisms. The latter hypothesis aligns more closely with the established bidirectional relationship between epilepsy and dementia [17].

One final observation regarding the limited characterization of DLB following LOEU diagnosis warrants emphasis. The diagnostic criteria for prodromal DLB are relatively new, so this disease stage may have been historically underrecognized and underreported due to insufficient diagnostic tools [18]. Future prospective studies tracking LOEU progression that incorporate these clinical criteria and ASyn-SAA testing when suggestive symptoms appear will undoubtedly clarify the prevalence of DLB among dementia syndromes affecting LOEU patients.

In conclusion, this case suggests that CSF ASyn-SAA should be included in the analysis panels of LOEU cases with features of MCI-LB in order to accurately assess their risk of progression to dementia, especially in dual-negative cases (i.e., those with no detectable amyloid and tau changes) [19]. This could be crucial if research identifies ASyn-targeted drugs with disease-modifying effects. This precision medicine approach would already allow for an earlier patient-centred strategy involving multidisciplinary teams (e.g., neurologists, psychiatrists, and geriatricians) to avoid potentially harmful medications (e.g., anticholinergics or neuroleptics), conduct regular neuropsychological and behavioral screening, provide targeted care for debilitating symptoms (e.g., cognitive decline, parkinsonism, hallucinations), and offer caregiver support.

## CRediT authorship contribution statement

**Augustin Moreau:** Writing – original draft, Formal analysis, Data curation. **Elisabeth Ruppert:** Writing – review & editing, Validation, Supervision, Methodology, Formal analysis, Data curation. **Frédéric Blanc:** Writing – review & editing, Supervision, Resources, Formal analysis. **Olivier BOUSIGES:** Writing – review & editing, Validation, Methodology, Investigation, Formal analysis, Data curation. **Benjamin Cretin:** Writing – review & editing, Validation, Supervision, Project administration, Methodology, Investigation, Formal analysis, Data curation, Conceptualization.

## Funding

The authors have no funding to report.

## Declaration of competing interest

The authors declare that they have no known competing financial interests or personal relationships that could have appeared to influence the work reported in this paper.

## Glossary

Aβ-42: amyloid peptide 1–42
AD: Alzheimer’s disease
ASyn: α-synuclein
BADL: basic activities of daily living
CSF: cerebrospinal fluid
CSF-ASyn-SAA: cerebrospinal fluid α-synuclein seed amplification assay
DLB: dementia with Lewy bodies
FP-CIT scan: [123I] N-x-flouropropyl-2b-carbomethoxy-3b-(4-iodophenyl) nortropane scan (DaT-scan)
IADL: instrumental activities of daily living
LCS: lacosamide
LOEU: late onset epilepsy of unknown etiology
MCI: mild cognitive impairment
MCI-LB: mild cognitive impairment with Lewy bodies
MMSE: minimental state examination
MRI: magnetic resonance imaging
PET: positron emission tomography
RBD: REM-sleep behavior disorder
REM-sleep: rapid eye movement sleep
V-EEG: video-electroencephalogram
